# Transient Expression of Lymphatic Markers in Retrobulbar Intraconal Orbital Vasculature During Fetal Development

**DOI:** 10.1167/iovs.61.6.22

**Published:** 2020-06-09

**Authors:** Quincy C. C. van den Bosch, Jackelien G. M. van Beek, Emine Kiliç, Robert M. Verdijk

**Affiliations:** 1 Department of Pathology, Erasmus MC University Medical Center, Rotterdam, Rotterdam, The Netherlands; 2 Department of Ophthalmology, Erasmus MC University Medical Center, Rotterdam, Rotterdam, The Netherlands; 3 Department of Ophthalmology, Albert Schweitzer Hospital, Dordrecht, The Netherlands

**Keywords:** lymphatics, transient expression, fetal development

## Abstract

**Purpose:**

The aim of this study is to investigate the presence of orbital lymphatic vessels during fetal and neonatal development and in adults using a panel of lymphatic markers.

**Methods:**

This was a retrospective observational case series. For analyzing lymphatic vessels, we used formalin-fixed paraffin-embedded enucleated eyes from 25 human fetuses between 13 and 24 weeks of gestation and postnatal eyes from 15 children and 5 adults. Immunohistochemical analysis of lymphatic vessels was performed for the markers: lymphatic vessel endothelial hyaluronic acid receptor-1 (LYVE-1), podoplanin (D2-40), Prospero-related homeobox gene-1 (Prox-1), pan-endothelial marker CD31, and blood vessel endothelium specific CD34.

**Results:**

Vasculature showing endothelial expression of LYVE-1, D2-40, Prox-1, and CD31 in combination with absence or weak expression of CD34, as would be expected for lymphatic vessels, was seen in 11 of 25 fetuses in an age range from 14 weeks to 23 weeks of gestation (44%). This lymphatic vascular staining pattern was also observed in 4 of 15 liveborn children (27%), all within 1 month of age, of which two were born prematurely at 32 and 34 weeks of gestation. Interestingly, an incomplete lymphatic staining pattern was observed in another 4 fetuses and two liveborn children of 4 months and 7 years old. No expression of lymphatic markers was observed in adult orbital vasculature.

**Conclusions:**

No retrobulbar intraorbital lymphatic vessels were observed in adults, however, we did observe transient expression of lymphatic markers in retrobulbar intraconal orbital vasculature during fetal and early neonatal development. The orbit may, therefore, be proposed to possess a full range of lymphatic plasticity.

The lymphatic system is important for transportation of immune cells, interstitial fluid balance, and macromolecular absorption (lipids).^1^ The organization of the lymphatic system generally parallels that of the blood vascular system, but lymphatics are not distributed as uniformly throughout the body.^1^ The eyelids and conjunctiva are rich in blood vessels and lymphatics, whereas hypo vascular tissues, such as the cornea,^2^ sclera,^3^ and lens, have none. Other well vascularized tissues, the central nervous system, the eye with exception to the conjunctiva and limbus, and orbit, also appear devoid of lymphatics.^4^^–^^7^

Lymphangiogenesis of the orbit has been investigated extensively.^8^ Previous studies of adult orbital soft tissues have reported that orbital fat lacks lymphatic vessels, but that inflammation can induce both the growth of new blood vessels and lymphangiogenesis in orbits that are inflamed^9^ or in orbital infection.^10^ It is thought that orbital soft tissues do not contain lymphatic vessels except for lymphatic-like structures around the dura mater surrounding the optic nerve and in the lacrimal gland.^11^^,^^12^ Whether “classical” orbital lymphatics exists, therefore, is controversial.

Lymphatic vessels are derived from venous endothelial cells during embryogenesis. Whether or when the presumed orbital lymphangiogenic privilege is achieved during development is not known yet. For the developing human, it is not known whether the fetal orbit is primarily alymphatic or if there is a regression of lymphatic vessels that may transiently evolve during embryogenesis, as has been described for the murine cornea.^13^

Lymphatic vessels can be identified using a panel of immunohistochemical markers.^14^^–^^17^ Endothelial markers recognize growth factors and differentiation antigens specific for lymphatic endothelial cells. It is recommended to use a minimum panel of three endothelial antibodies, which consists of one pan-endothelial marker and at least two different lymphatic endothelial-specific antibodies, to avoid misinterpretation of staining results.^14^^,^^16^ Podoplanin (D2-40) is a mucin-type transmembrane glycoprotein, expressed in lymphatic endothelial cells and other cells, such as macrophages and tumor cells.^1^^,^^18^ Its function includes regulation of lymphatic vascular formation and platelet aggregation. D2-40 is the most commonly used mouse monoclonal antibody against Podoplanin. Prospero-related homeobox gene-1 (Prox-1) is a nuclear transcription factor and of key importance for the development of the lymphatic system. Prox-1 is also expressed in nonendothelial cell types, such as hepatocytes, bile duct epithelium, pancreatic epithelium, cardiomyocytes, lens, retina, spinal ganglia, and vegetative ganglia.^19^^,^^20^ It is not expressed in blood vascular endothelial cells, except for a small segment of the anterior cardinal vein.^21^ Knockout models of Prox-1 discovered the crucial role of Prox-1 in the development of the lymphatic system.^22^ Lymphatic vessel endothelial hyaluronic acid receptor-1 (LYVE-1) is an integral membrane glycoprotein and lymphatic vessel endothelial hyaluronan receptor type 1. LYVE-1 is expressed in lymphatic, but not in blood vascular endothelium.^1^ LYVE-1 may also be expressed by activated macrophages.^23^ CD-31 (platelet endothelial cell adhesion molecule-1, PECAM) is the most sensitive and specific pan-endothelial marker. It is an integral membrane glycoprotein, expressed on endothelial intercellular junctions, but may also be expressed by macrophages.^24^ CD-34 is a single-chain transmembrane glycoprotein, a hematopoietic progenitor cell antigen, and is expressed in the endothelial cells of blood vessels, but not of non-neoplastic lymph vessels.^25^ CD34 may also be expressed by stromal fibroblasts.^26^ Because all these markers may also be expressed in other cells than lymphatic endothelium, it is difficult to identify a lymphatic vessel based on a single marker. When multiple markers are used, lymphatic vessels will be identified with increased accuracy.

In this study, the expression of lymphatic markers is examined in the retrobulbar intraconal orbit of the developing and adult human eye with emphasis on maturation dependent changes and the possible presence of lymphatic structures within the orbit.

## Methods

### Sample Selection

Human eyes were obtained by enucleation from termination of pregnancy fetuses between gestational weeks 13 and 24 (*n* = 25), deceased children between the ages of 0 and 15 years of age (*n* = 15), and adults (*n* = 5) as part of routine diagnostic procedures. All studies complied with the regulations of the local ethics committee. Clinical information and gestational age of fetuses and ages of the children and adults are provided in Table. Because prenatal ultrasound examination showed congenital malformations, pregnancy was terminated. The fetal eyes were harvested in case of brain malformations or with a differential diagnosis that involved syndromes that may be associated with ocular malformations. None of the diagnoses involved syndromes associated with (lymphatic) vascular malformations, like Turner-, Proteus-, Sturge-Weber-, or Klippel-Trenaunay-Weber syndrome. None of the eyes did show developmental anomalies upon macroscopic and microscopic evaluation. None of the liveborn children or adults suffered orbital infectious or inflammatory disease.

**Table.** Description of the Cases With Causes of Death

Case No.	Age	Cause of Death/Abortion	LYVE-1	Prox-1	D2-40	CD31	CD34
1	13 + 3 wk	Omphalocele and encephalocele	+	+	−	+	N/A
2*	14 + 0 wk	Steinfeld syndrome	+	+	+	+	w
3	16 + 1 wk	Osteogenesis imperfecta type II	+	+	+	+	−
4	16 + 4 wk	Holoprosencephaly with MCA	−	−	−	+	+
5	16 + 5 wk	Arthrogryposis	+	+	+	+	−
6	17 + 0 wk	Joubert syndrome	−	−	−	−	−
7	18 + 5 wk	Isolated lumbosacral myelomeningocele	+	+	+	+	−
8*	18 + 6 wk	Premature rupture of membranes	+	+	+	+	−
9	21 wk	Ellis van Creveld syndrome	−	−	−	+	+
10	21 wk	MCA no syndrome diagnosis	+	+	+	+	N/A
11*	21 + 2 wk	MCA no syndrome diagnosis	+	+	+	+	w
12	21 + 3 wk	Aqueductal stenosis	+	+	+	N/A	N/A
13	21 + 4 wk	Aqueductal stenosis	+	+	+	+	_
14	21 + 4 wk	Osteogenesis imperfecta	−	−	−	+	+
15	22 + 4 wk	Vermis and callosal hypoplasia	−	−	−	+	+
16	22 + 4 wk	Walker Warburg syndrome	−	−	−	+	+
17	22 + 5 wk	Isolated corpus callosum agenesis	+	+	+	+	−
18	22 + 6 wk	IUGR due to maternal pre-eclampsia	−	−	−	+	+
19	23 + 0 wk	MCA no syndrome diagnosis	+	+	+	+	−
20	23 + 1 wk	Isolated ventriculomegaly	−	−	−	+	+
21	23 + 3 wk	TUBB2B gene mutation	+	+	+	+	−
22	23 + 3 wk	Unexplained hydrops foetalis	+	+	+	N/A	N/A
23	23 + 5 wk	Isolated complex cardiac malformation	+	+	+	+	−
24	24 wk	Isolated midline arachnoidal cyst	−	−	−	+	+
25*	24 + 3 wk	Diaphragmatic hernia	−	−	−	+	+
26	27 + 2 wk 6 d old	Perinatal death, Goldenhar syndrome	−	−	−	+	+
27	32 + 6 wk 2 d old	Perinatal death, diaphragmatic hernia	+	+	+	+	−
28	33 + 5 wk 5 d old	Perinatal death, lissencephaly spectrum	−	−	−	+	+
29#	34 + 5 wk	Hydrocephalus	+	+	+	+	−
30	1 day old	Perinatal death, abusive head trauma	+	+	+	+	−
31	8 days old	Chondrodysplasia punctate	+	+	+	+	−
32	6 wk	Abusive head trauma	−	−	−	+	+
33	7 wk	Abusive head trauma	−	−	−	+	+
34	4 mo	Abusive head trauma	−	−	−	+	+
35	4 mo#	Hemophagocytic lymphohistiocytosis	+	−	+	+	+
36	6 mo	Pneumonia	−	−	−	+	+
37	7 mo	Abusive head trauma	−	−	−	+	+
38	12 mo	Endomyocarditis	−	−	−	+	+
39	7 y#	Bronchopneumonia	+	−	−	+	+
40	15 y	Ketoacidosis	−	−	−	+	+
41	32 y	Decompensatio cordis	−	−	−	+	+
42#	53 y	Choroidal melanoma	−	−	−	+	+
43	54 y	Unknown (eye bank specimen)	−	−	−	+	+
44	74 y	Bronchopneumonia	−	−	−	+	+
45	68 y	Bronchopneumonia	−	−	−	+	+

The last five columns represent the staining pattern per individual case. Cases that could not be investigated using the full panel of markers due to lack of tissue, technical issues during staining or couldn't be interpreted with confidence are indicated as not assessable (N/A).

**Figure 1.**
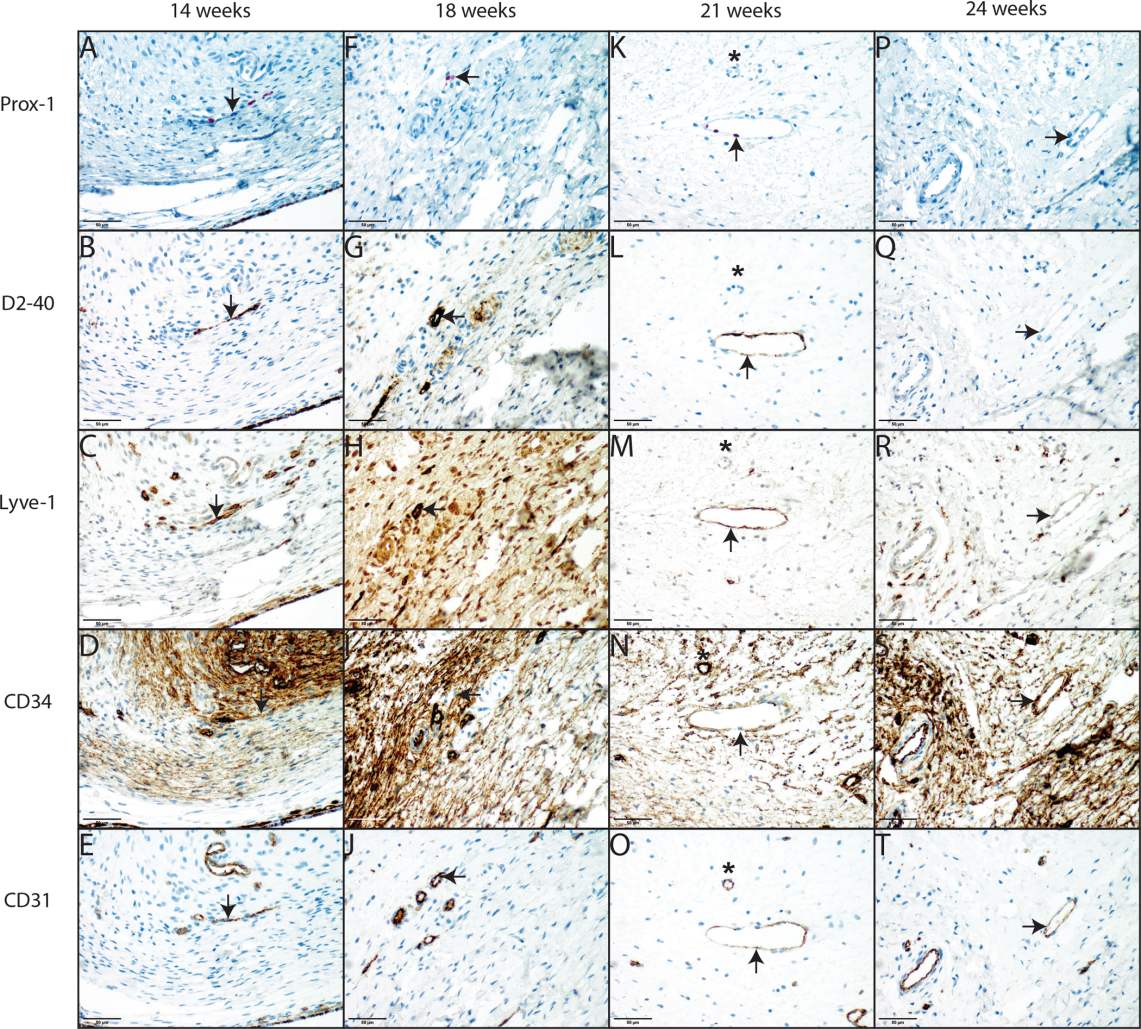
Overview of identification of lymphatic vessels with Prox-1, D2-40, LYVE-1, CD34, and CD31 in second trimester fetuses. Lymphatic phenotype staining pattern of a 14-week-old fetus (Table, case 2) is shown by a positive endothelial staining for Prox-1, D2-40, and LYVE-1 (**A,** **B,** **C**, respectively) combined with a weaker staining for CD34 compared to the surrounding blood vessel endothelium. (**D**) Positive staining for CD31. (**E**) Lymphatic staining pattern of an 18-week-old fetus (Table, case 8) showed positive endothelial phenotype staining for Prox-1, D2-40, and LYVE-1 (**F,** **G,** **H**) combined with a negative staining for CD34 (**I**) and a positive staining for CD31. (**J**) Lymphatic phenotype staining pattern of a 21-week-old fetus (Table, case 11) showed positive endothelial staining for Prox-1, D2-40, and LYVE-1 (**K,** **L,** **M**, respectively) combined with a weak staining for CD34. (**N**) and a positive staining for CD31. Note the asterisk (*) showing strong positive staining in blood vessel endothelium for CD34 as reference. (**O**) Staining pattern of vasculature in a 24-week-old fetus (Table, case 25) showed negative staining for Prox-1, D2-40, and LYVE-1 (**P,** **Q,** **R**, respectively) combined with a positive staining for CD34 (**S**) and a positive staining for CD31. (**T**) No lymphatic phenotype staining pattern was observed in this case.

**Figure 2.**
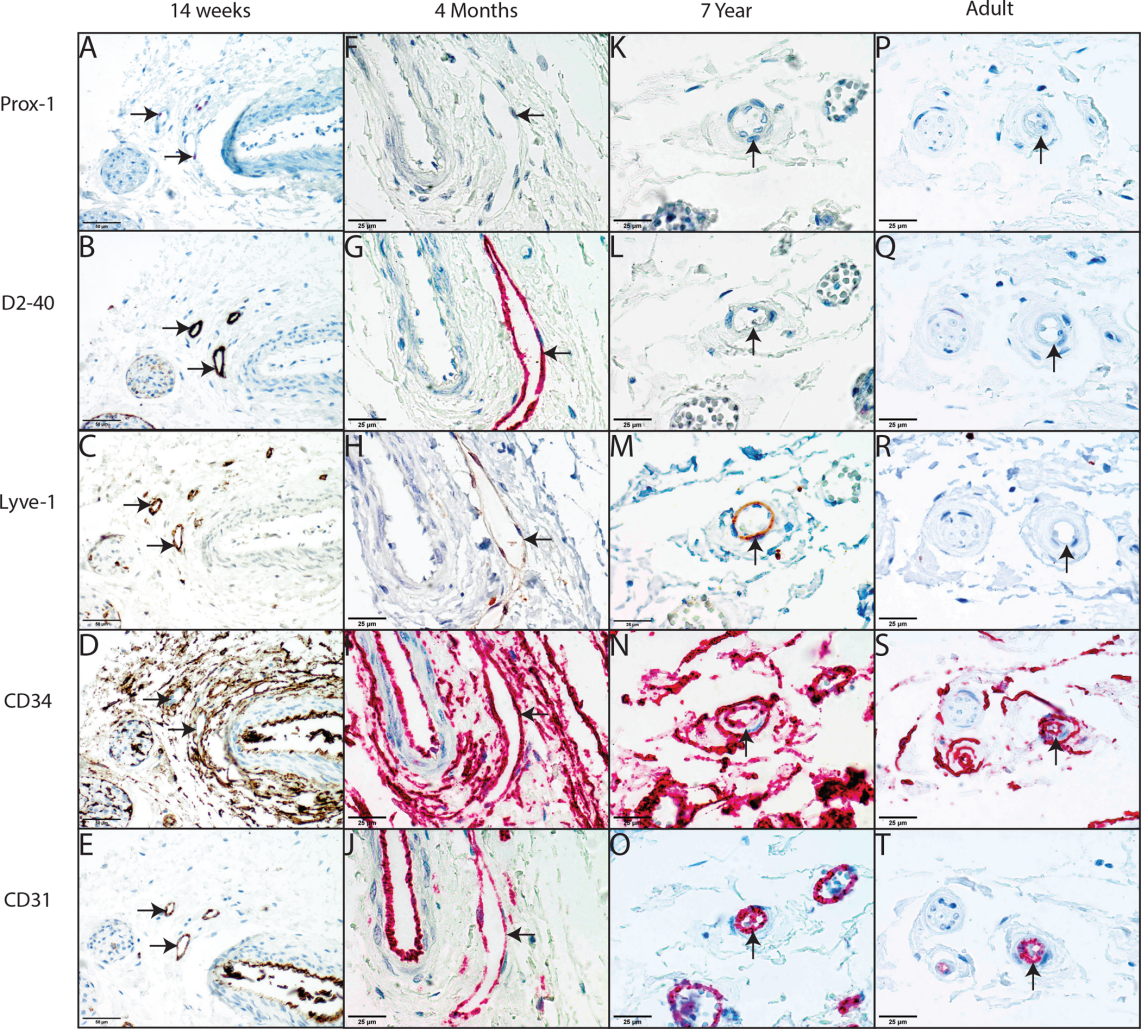
Overview of identification of lymphatic vessels with Prox-1, D2-40, LYVE-1, CD34, and CD31 in third trimester and older children and adult. Lymphatic phenotype staining pattern of a 14-week-old premature born child at 34 weeks of gestation (Table, case 29) is shown by a positive staining for Prox-1, D2-40, and LYVE-1 (**A,** **B,** **C**, respectively) combined with a negative staining for CD34 (**D**) and a positive staining for CD31. (**E**) Incomplete lymphatic staining pattern of a 4-month-old child showed positive staining for LYVE-1 and D2-40 (**G,** **H**), but a negative staining for Prox-1. (**F**) Whereas CD34 and CD31 both show positive staining. (**I,** **J**) Incomplete lymphatic staining pattern of a 7-year-old child showed positive staining for LYVE-1 (**M**), but negative staining for Prox-1 and D2-40. (**K,** **L**) CD34 and CD31 both showed positive staining. (**N,** **O**) Vascular staining pattern of an adult showed no positive staining of Prox-1, D2-40, and LYVE-1 (**P,** **Q,** **R**), but did show positive staining for CD34 and CD31. (**S,** **T**) No lymphatic phenotype staining pattern was observed in adult cases.

The cases illustrated in Fig. 1 have been highlighted with an *, the cases illustrated in Fig. 2 have been highlighted with an #. One adult case was obtained due to enucleation of uveal melanoma.

IUGR = intrauterine growth retardation; MCA = multiple congenital malformations; w: weak expression.

Eyes were fixated in buffered 10% formaldehyde, and pupil-optic nerve sections of 4 mm thickness were obtained after paraffin embedding. Pupil-optic nerve sections were stained for hematoxylin & eosin (H&E), and the presence of sufficient retrobulbar orbital fat for evaluation was confirmed. In addition, immunohistochemistry was performed on serial pupil-optic nerve sections.

### Immunohistochemistry

Formalin-fixed paraffin-embedded (FFPE) sections were analyzed for the presence of lymphatic vessels. Four-micrometer thick sections were stained for Podoplanin (Clone D2-40, Ref.: 760-4395; Cell Marque, Rocklin, CA, USA), Prospero Homeobox-1 (Prox-1, Clone D2J6J, dilution 1:1500; Cell Signaling, Leiden, The Netherlands), Cluster Differentiation 31 (CD31, Clone JC70, Ref.: 760-4378; Cell Marque, Rocklin, CA, USA), and Cluster Differentiation 34 (CD34, Clone QBEnd/10, Ref.: 790-2927; Ventana Medical Systems, Tucson, AZ, USA) with the Ventana Benchmark Ultra automated staining system (Ventana Medical Systems). Briefly, after deparaffination the sections were processed for 32 to 64-minute antigen retrieval using Cell Conditioning Solution 1 (CC1 Ventana Ref.: 950-124). Following 32-minute incubation (16-minute for CD31) with the primary antibody at 36 deg Celsius (°C), detection was performed using the ultraView Universal Alkaline Phosphatase Red Detection Kit (Ref.: 760-501; Ventana Medical Systems) in combination with the Amplification Kit (Ref.: 760-080; Ventana Medical Systems). Sections were counterstained with hematoxylin II (Ref.: 790-2208; Ventana Medical Systems). Due to technical limitation of the Ventana automated staining system, detection of LYVE-1 required manual interference in the protocol. For anti-LYVE-1 (Clone AF2089, dilution 1:1000; R&D Systems, Minneapolis, MN, USA) primary antibody staining was executed using the Ventana Discovery Benchmark automated staining system (Ventana Medical Systems). The following adaptations from the protocol were required: endogenous peroxidase was blocked in order to prevent unspecific signal using Inhibitor CM from the DISCOVERY ChromoMap DAB Kit (RUO) (Ref.: 760-159; Ventana Medical Systems) for 4 minutes. The secondary antibody incubation was performed with anti-Goat-HRP (Ref.:760-159; Ventana Medical Systems) for 32 minutes. Detection was executed manually with 3-amino-9-ethylcarbazoledue (AEC) diluted in 0.2M Sodium Acetate with H_2_O_2_. The slides were counterstained with Mayer's Hematoxylin (Cat. 4085.9005; Klinipath, Duiven, The Netherlands).

### Scoring of Immunohistochemistry

Three independent reviewers scored the slides to reach consensus: a pathologist, an ophthalmologist, and a research technician with ample experience in ophthalmic pathology. Based on the presence or absence of specific signals in the orbital vasculature, lymphatic vessels were identified using a panel of immunohistochemical markers on serial sections. As described in an earlier study, a vascular structure was identified as a lymph vessel when it showed combined endothelial expression of D2-40, Prox-1, LYVE-1, and CD31 and absence or weak expression of CD34.^17^ These requirements are in accordance with the first international consensus on the methodology of lymphangiogenesis quantification in solid human tumors and the consensus statement on the immunohistochemical detection of ocular lymphatic vessels.^14^^,^^16^ Lymphatic vessels of the perilimbal conjunctiva served as internal controls, external control tissue (lymph node) was applied in case of absence of conjunctival tissue.

## Results

### Immunohistochemistry for Endothelial Lymphatic Markers

Positive endothelial staining for any of the lymphatic vascular markers was observed in the retrobulbar perioptic orbital fat of fetuses and young children. None of the adult cases showed positive endothelial staining for lymphatic markers, which is in concurrence with earlier unreported observations in enucleation specimen for uveal melanoma.^17^ Positive staining for all lymphatic markers tested in combination with weak or absence of staining for CD34 was seen in 11 of 25 fetuses in an gestational age ranging from 13 to 24 weeks (44%) (Table, Fig. 1). In another three fetuses, CD34 staining could not be evaluated with certainty because of high positive staining of perivascular orbital connective tissue. This could potentially have resulted in an underestimation of the maximum total number of positive cases (56%), see Table. Furthermore, this lymphatic vascular phenotype was observed in 4 of 15 liveborn children (27%), all within 1 month of age, of which 2 were premature born at 32 and 34 weeks of gestation, respectively (Table, Fig. 2). The number of vascular structures that showed a lymphatic vascular phenotype varied from one to six in each case. Often a symmetrical pattern was observed showing positivity both at the nasal and temporal side of the optic nerve.

### Transient Expression of Lymphatic Markers in Relation to Age

Three cases, one fetus of 13 weeks of gestation, one term born, one 4-month-old child, and one 7-year-old child, showed a vascular structure that exhibited incomplete lymphatic endothelial marker staining (Table). The 13 weeks of gestation fetus with a vascular structure staining positive for LYVE1, Prox1, and CD31 was a termination of pregnancy because of the combination of omphalocele and encephalocele. CD34 could not be evaluated in this case. The 4-month-old child with a vascular structure positive for LYVE-1, D2-40, CD31, and CD34 died of hemophagocytic lymphohistiocytosis, which was not present in the orbital tissues. The 7-year-old child had a vascular structure that stained positive for LYVE-1 in combination with CD31 and CD34 positive staining. This implies a transient expression of lymphatic markers and that Prox-1 may the first marker to be lost followed by D2-40 and LYVE 1. Moreover, for second trimester fetuses, a lymphatic vascular pattern was observed in a maximum of 14 of 25 cases (56%, including those cases where CD34 could not be evaluated), whereas in third trimester premature and term born children, such a complete pattern was only observed in 4 of 15 (27%) cases. No lymphatic vascular patter was found in adults, also indicating a progressive loss of expression of lymphatic markers with age.

## Discussion

This study examined the presence of retrobulbar intraorbital lymphatic vessels during fetal and neonatal development and in adults by using a panel of lymphatic markers on enucleation specimen. Our panel of markers was designed to distinguish lymphatic vessels from blood vessels. Our criteria to identify lymphatic vessels also relies on the absence or weak expression of CD34, which should be carefully interpreted in early development and adulthood. During early development, CD34 is expressed in mesenchymal cells,^26^ hematopoietic progenitor cells,^28^ and developing vasculature.^29^ Furthermore, endothelial cell-fate during early development is much less understood, which makes identification of lymphatic vessels challenging in embryonic tissue. Whereas in adulthood, CD34 can be occasionally and irregularly expressed in lymphatic vessels depending on the type of vessel, localization, and tissue, albeit in a much lower staining intensity when compared to blood vessels.^30^^,^^31^ When identifying lymphatics in neural-rich tissue, such as the eye, one needs to be aware of positive staining of D2-40^32^ and CD34^33^ in nerve sheath cells. Especially in an immunofluorescent approach, this can mimic a vascular structure, and, thus, may appear as a lymphatic vessel. With the use of a broader lymphatic marker panel, these structures can be identified as nonlymphatic structures by lack of LYVE-1, Prox-1, and CD31 (data not shown). We could not detect retrobulbar intraorbital lymphatic vasculature in adult samples using five specific markers. This is in concordance with earlier unreported observations, when 16 adult eyes were examined for lymphatic vessel recruitment in uveal melanoma.^17^ However, we did find transient expression of lymphatic markers in retrobulbar orbital vasculature during fetal and early neonatal development. Others did not observe intraorbital lymph vessels in four 10 to 12 weeks of gestation old fetuses that were serially sectioned.^27^ Although, in that study, only podoplanin was used as an immunohistochemical marker and our cases are of more advanced gestational age. The use of multiple markers in the current study may explain the increased likelihood for identification of lymphatic vasculature. The fact that not all cases from a similar gestational age proved to be positive may be explained by a potential for under detection because a limited amount of orbital fat was available in the enucleation specimen. Complete removal, embedding, and sectioning of the orbital contents may be expected to increase the likelihood for identification of such vessels in future. Because such a procedure would not be justified as part of a diagnostic procedure, a separate specific parental consent would be required. The observation that, in the current study, expression of lymphatic markers decreases with (gestational) age, made us hypothesize that this is most likely a transient developmental phenomenon that does not signify consistent orbital lymphangiogenesis. These findings are similar to what has been reported for the murine cornea.^13^ That study demonstrated that the mouse cornea was endowed with a significant number of lymphatic vessels that underwent spontaneous formation and regression during a critical period after birth, which was not observed for blood vessels. Lymphatic growth can be reactivated in the adult cornea and orbit after inflammatory stimulation. The transient expression of lymphatic vessel markers in retrobulbar orbital vessels during fetal and early neonatal development may, therefore, share overlapping features with lymphatic vessel growth and regression during postnatal development in the mouse model. In line with what was described for the murine cornea, it could be speculated that the lymphatic status of the orbit is orchestrated and maintained by a similar combination of pro- and antilymphatic factors already known or yet to be discovered. Certain physiologic or pathologic stimulations will tip the balance in favor of lymphatic formation or regression.^13^ This may also explain previous studies of adult orbital soft tissues that have reported that orbital fat lacks lymphatic vessels, but that inflammation can induce both the growth of new blood vessels and lymphangiogenesis in orbits that are inflamed or in orbital infection.^9^^,^^10^

In conclusion, this study describes that transient developmental expression of lymphatic markers is a feature observed in retrobulbar intraconal vasculature during the fetal and early neonatal period. The orbit may, therefore, be proposed to possess a full range of lymphatic plasticity.
